# Adoption of a novel biomarker-guided quality improvement treatment bundle for patients with subclinical acute kidney injury after cardiac surgery: An implementation study

**DOI:** 10.1097/EJA.0000000000002315

**Published:** 2025-11-21

**Authors:** Benjamin Milne, Sinead Helyar, Carol Pellowe, Marlies Ostermann, Nicholas Lees, Jackie Donovan, Mandeep Sekhon, Gudrun Kunst

**Affiliations:** From the The Department of Anaesthesia, King's College Hospital NHS Foundation Trust, London, UK (BM, GK), the Department of Critical Care, King's College Hospital NHS Foundation Trust, London, UK (BM), NIHR Academic Clinical Fellow, King's College London, London, UK (BM), ACET Research Team, King's College Hospital NHS Foundation Trust, London, UK (SH), the Royal College of Anaesthetists Lay Committee (CP), the Department of Critical Care, Guy's & St Thomas’ NHS Foundation Trust, London, UK (MO), the Department of Critical Care, Royal Brompton & Harefield Hospitals, Guy's & St Thomas’ NHS Foundation Trust, London, UK (NL), Clinical Biochemistry, Royal Brompton & Harefield Hospitals, Guy's & St Thomas’ NHS Foundation Trust, London, UK (JD), Population Health Research Institute, School of Health and Medical Sciences, City St George's, University of London, London, UK (MS), the School of Cardiovascular and Metabolic Medicine and Sciences, King's College London, London, UK (GK)

## Abstract

**BACKGROUND:**

Evidence-based peri-operative practices can deliver improved patient outcomes, but their benefits may be limited by obstacles to implementation into routine practice. A multicentre randomised controlled trial demonstrated a reduction in the incidence of moderate-severe acute kidney injury after cardiac surgery using a biomarker-guided renal care bundle, however these measures are not a routine part of clinical practice.

**OBJECTIVE:**

We set out to assess the key implementation domains of acceptability, feasibility and appropriateness of this technology-intervention bundle in the cardiac intensive care unit.

**DESIGN:**

Following introduction of the biomarker-guided renal care bundle, the implementation process was assessed using comprehensive and novel implementation science metrics: Weiner's adoption metrics and Sekhon's theoretical framework of acceptability.

**SETTING:**

Two tertiary cardiac surgical centres in London, UK, between July 2021 and February 2022.

**PARTICIPANTS:**

One hundred and seventy-six adult patients underwent urinary biomarker assessment 2 h after arrival on the cardiac intensive care unit and 49 respondents, including medical and surgical consultants and nursing staff, completed our implementation survey.

**INTERVENTIONS:**

Patients with a raised biomarker level ([TIMP-2] × [IGFBP7] > 0.3 (ng ml^−1^)^2^ 1000^−1^) received a renal care bundle. The clinician respondents underwent a survey designed to provide implementation metrics.

**MAIN OUTCOME MEASURE:**

The primary outcome was the assessment of implementation using Weiner's adoption metrics and Sekhon's theoretical framework of acceptability.

**RESULTS:**

34.7% (*n* = 61) of patients had a raised biomarker level indicating a requirement for the renal care bundle, with a mean delivery of 4.4 (out of 6) bundle items. Concerning Weiner's adoption metrics, the median scores for acceptability, appropriateness and feasibility were all 4 (out of a possible 5) indicating a high degree of positivity towards the intervention. Similarly, all domains of Sekhon's theoretical framework of acceptability scored a median of 4 (out of 5). The highest scoring domain was Perceived Effectiveness (mean 4.0), whilst the lowest were Burden (mean 3.4) and Opportunity Costs (mean 3.4).

**CONCLUSIONS:**

This implementation science study demonstrates that this biomarker-guided renal care bundle is deliverable after cardiac surgery, outside of a research setting. Our findings show that the intervention is acceptable, feasible and appropriate, which is strongly supported by its ‘perceived effectiveness’. We have also demonstrated the more negative aspects to be addressed to improve concordance in other settings.

**KEY POINTS**

- Evidence-based biomarker-guided renal care bundles have been shown to reduce the incidence and severity of acute kidney injury after cardiac surgery.
- Despite the evidence, studies have shown restricted uptake into routine clinical practice.
- This technology-intervention bundle is deliverable outside of strict research settings, increasing the delivery of renal protective measures.
- Despite potentially increasing staff workload, the practice scored highly in terms of acceptability, feasibility and appropriateness.
- Staff were likely to be influenced by the perceived effectiveness of the intervention, whilst improving uptake should focus on reducing the additional workload and the opportunity costs it may represent.

## Introduction

Implementation of evidence-based practices in medicine, and specifically in anaesthesia and peri-operative medicine, has been demonstrated to be a challenge with poor uptake of clinical practice guidelines.^1–3^ Indeed, the estimated time from new scientific discoveries until patient benefit has been described as taking 17 years.^4^ Implementation Science in peri-operative medicine is relatively novel.^5^ Improvement metrics serve to provide evidence of key factors such as acceptability, feasibility and appropriateness, which may provide justification for the costs often associated with innovation, and these have been reviewed recently.^6^

In this implementation study, we assess the adoption of a novel biomarker-guided quality improvement treatment bundle for patients with subclinical acute kidney injury (AKI) after cardiac surgery by clinical teams (ADOPT-QI-AKI). We introduce two metrics into peri-operative medicine: a broad and easily applicable adoption metrics test, the Weiner Adoption Metrics (WAM) test, which considers three domains (acceptability, appropriateness and feasibility) and the novel comprehensive Theoretical Framework of Acceptability Questionnaire (TFA-Q) test, investigating seven different domains (affective attitude, burden, ethicality, perceived effectiveness, intervention coherence, self-efficacy, opportunity costs) and general acceptability.^7,8^

AKI affects 22 to 36% of patients after cardiac surgery, contributing to mortality and morbidity.^9–14^ Given this burden of disease, strategies to prevent and mitigate AKI are much sought after.^15^ Enhanced Recovery After Surgery (ERAS) is an evidence-based care improvement process for surgical patients.^16^ The guidance for Enhanced Recovery After Cardiac Surgery (ERACS) has prioritised the reduction of AKI, including the recommendation to use AKI biomarkers for early identification of patients at risk of AKI and to guide an intervention strategy to reduce renal dysfunction (a Class IIa, Level B recommendation).^17,18^ In cardiac surgery, the elevation of combination of two biomarkers of urinary cell cycle arrest (tissue inhibitor metalloproteinase 2 (TIMP-2), and insulin like growth factor binding protein 7 (IGFBP7)) expressed as ([TIMP-2] × [IGFBP7]) has been shown to be useful to predict any stage of AKI at 4 h after cardiopulmonary bypass (CPB) and its use in combination with a biomarker-guided treatment bundle reduced the incidence of AKI in recent single and multicentre studies.^19–21^ However, this test and the associated treatment bundle are not routinely used in cardiac surgical patients.

We set out to assess the implementation of this AKI biomarker-guided renal care bundle to treat subclinical AKI after cardiac surgery, determining acceptability, feasibility and appropriateness of this technology-intervention bundle using comprehensive and novel implementation science metrics (WAM and TFA tests) in two tertiary cardiac surgery centres.

## Methods

Ethical approval for this study was not required because it was deemed a quality improvement innovation and implementation project. It was granted local institutional approval (RBH KHP AKI-CAR008).

We undertook a baseline survey of local clinical practice for management of cardiac postoperative care, assessing existing penetrance of any aspects of the proposed evidence-based KDIGO (Kidney Disease Improving Global Outcomes) bundle intervention. Following this we introduced our biomarker-guided renal care bundle sequentially in two tertiary cardiac centres in London, UK.

Medical and nursing staff on the cardiac intensive care units (CICU) were trained in the use of an automated immunofluorescence assay for measurement of urinary concentrations of tissue inhibitor metalloproteinase 2 (TIMP-2) and insulin -like growth factor binding protein 7 (IGFBP7) (NephroCheck, BioMerieux SA, France & Astute140 Meter, Astute Medical Inc., USA) and in the delivery of the renal care bundle. Following this training period, 100 patients were scheduled to undergo urinary biomarker evaluation 2 h after admission to the CICU in each centre. Patients with urinary [TIMP-2] × [IGFBP7] concentration > 0.3 (ng ml^−1^)^2^ 1000^−1^ were eligible for receipt of the KDIGO-based renal care bundle including 6 interventions: discontinuation of nephrotoxic drugs, use of advanced haemodynamic monitoring and goal directed interventions, close monitoring of renal function, avoidance of hyperglycaemia, discontinuation of Angiotensin-Converting Enzyme inhibitors (ACEi) and Angiotensin Receptor Blockers (ARBs) for 48 h, and avoidance of hydroxyethyl starch (HES) and gelatin or chloride-rich solutions (Table 1, Supplemental Digital Content).^20,21^ Following the training period, the incorporation of routine urinary biomarker testing and delivery of the care bundle were managed by the clinical staff, without the supervision of research medical and nursing staff. A maximum average rate of recruitment of 2.8 patients per day was anticipated.

As part of a quality improvement innovation and implementation project, the inclusion criteria were broad, accounting for all adult cardiac surgical patients undergoing a primary (i.e. not an urgent/emergency re-look/re-procedure) cardiac procedure with direct admission to CICU postoperatively. We only sought to exclude patients with KDIGO stage 5 chronic kidney disease or undergoing cardiac transplant. Patient outcomes were collected at 30-days following cardiac surgery.

Following conclusion of the study period, CICU medical and nursing staff were surveyed (using an online questionnaire) to collect the implementation-Science measures using WAM (Table 1) and Sekhon's TFA (Table 2), which served as the primary outcome measures.^7,8,22^

**Table 1:** Weiner adoption metrics (WAM) incorporating the domains of Acceptability of Intervention Measure (AIM), Intervention Appropriateness Measure (IAM) and Feasibility of Intervention Measure (FIM). Each statement received a graded response

Domain	Statement	Responses
Acceptability of Intervention Measure (AIM)	Urinary biomarker-guided KDIGO interventions meet my approval	1 – Completely disagree
	Urinary biomarker-guided KDIGO interventions are appealing to me	2 – Disagree
	I like urinary biomarker-guided KDIGO interventions	3 – Neither agree nor disagree
	I welcome urinary biomarker-guided KDIGO interventions	4 – Agree
Intervention Appropriateness Measure (IAM)	Urinary biomarker-guided KDIGO interventions seem fitting	5 – Completely agree
	Urinary-biomarker-guided KDIGO interventions seem suitable	
	Urinary biomarker-guided KDIGO interventions seem applicable	
	Urinary biomarker-guided KDIGO interventions seem like a good match	
Feasibility of Intervention Measure (FIM)	Urinary biomarker-guided KDIGO interventions seem implementable	
	Urinary biomarker-guided KDIGO interventions seem possible	
	Urinary biomarker-guided KDIGO interventions seem doable	
	Urinary biomarker-guided KDIGO interventions seem easy to use	

KDIGO, Kidney Disease: Improving Global Outcomes.

**Table 2:** Sekhon's Theoretical Framework of Acceptability Questionnaire (TFA-Q) and the constructs assessed. Each question or statement received a graded response

Question/statement	Response	
**Affective attitude**		
How comfortable do you feel in using the urinary biomarker test and renal care bundle?	1	Very uncomfortable
	2	Uncomfortable
	3	No opinion
	4	Comfortable
	5	Very comfortable
**Burden** ^a^		
How much effort will it take to use the urinary biomarker test and renal care bundle?	1	No effort at all
	2	A little effort
	3	No opinion
	4	A lot of effort
	5	Huge effort
**Ethicality** ^a^		
There are negative moral or ethical consequences to the use of the urinary biomarker test and renal care bundle as part of the clinical service	1	Strongly disagree
	2	Disagree
	3	No opinion
	4	Agree
	5	Strongly agree
**Perceived effectiveness**		
The urinary biomarker test and renal care bundle will help improve patient outcomes as part of the cardiac clinical service	1	Strongly disagree
	2	Disagree
	3	No opinion
	4	Agree
	5	Strongly agree
**Intervention coherence**		
It is clear to me how the urinary biomarker test and renal care bundle will help improve avoidance of acute kidney injury	1	Strongly disagree
	2	Disagree
	3	No opinion
	4	Agree
	5	Strongly agree
**Self efficacy**		
How confident did you feel about using the urinary biomarker test and renal care bundle?	1	Very unconfident
	2	Unconfident
	3	No opinion
	4	Confident
	5	Very confident
**Opportunity costs** ^a^		
The urinary biomarker test and renal care bundle will interfere with my other priorities	1	Strongly disagree
	2	Disagree
	3	No opinion
	4	Agree
	5	Strongly agree
**General acceptability**		
How acceptable is the urinary biomarker test and renal care bundle?	1	Completely unacceptable
	2	Unacceptable
	3	No opinion
	4	Acceptable
	5	Completely acceptable

a Scales reversed for analysis.

WAM outcome measures are designed to assess three domains of implementation; acceptability, feasibility and appropriateness.^7^ Within this context these outcomes can be defined as:^7,23^

(1) Acceptability – the perception amongst implementation stakeholders that the intervention is satisfactory.
(2) Feasibility – the degree to which the intervention can be successfully delivered within a specified setting.
(3) Appropriateness – the perceived compatibility of the intervention for a specific setting.

The survey questions (and scoring) for WAM are shown in Table 1.

The median [IQR) and mean ± SD values were calculated for each aspect of the AIM, IAM and FIM, as well as for a composite average score of each domain. A score of >3 indicates a degree of agreement and >4 a high degree of agreement. Conversely, a score <3 suggests disagreement, and profound disagreement <2.

The TFA is a research tool developed to further consider the key factor of acceptability in design, evaluation and implementation of healthcare interventions. Within this conceptual framework, acceptability is a multifaceted construct which reflects how people delivering or receiving an intervention consider it to be appropriate, dependent on experience-based cognitive and emotional responses to it. Using this framework, acceptability is considered within separate constructs:^8^

(1) Affective attitude – how an individual feels about the intervention.
(2) Burden – the amount of effort required to participate in the intervention.
(3) Ethicality – the extent to which the intervention has a good fit with an individual's value system.
(4) Perceived effectiveness – the extent to which the intervention has achieved its intended purpose.
(5) Intervention coherence – the extent to which the participant understands how the intervention works.
(6) Self-efficacy – the participants confidence that they can perform behaviour required to participate in the intervention.
(7) Opportunity costs – the benefits, profits or values that were given up to engage in the intervention.

The TFA has an accompanying questionnaire (TFA-Q) in which each construct is assessed by one question (measured by a 5-point Likert scale), with a final question relating to overall acceptability, which also acts as an assessment of the content validity (Table 2).

The questions relating to Burden, Ethicality and Opportunity Costs had their scales reversed for analysis, to homogenise the numerical ratings (i.e. to ensure that the most favourable response to the intervention scored a ‘5’ and the least favourable a ‘1’ where the original question and scale may have been assessing concordance to an unfavourable statement).

To assess each construct, the mean ± SD and median [IQR] responses to the TFA questionnaire were calculated. An average composite score of the seven constructs (the addition of the scores of all seven domains, before dividing by 7) was calculated, where a higher score indicates greater acceptability. Similar to the WAM, for the purposes of this study, a score >3 indicates a positive response (and 4 or more a high degree of positivity), whereas <3 indicates a negative response (and strongly negative at 2 or less).

To understand the relationship between the individual constructs of acceptability and both the composite and the general acceptability score, Spearman's correlation coefficient was calculated (and plotted graphically) (with *R* values of 0.00 to 0.19 considered very weak, 0.20 to 0.39 considered weak, 0.40 to 0.59 considered moderate, 0.60 to 0.79 as strong and 0.80 to 1.00 considered very strong).

Further assessments were made (using Spearman's correlation coefficient) of:

(1) Content validity – by assessing correlation between composite average score and the general acceptability, to see whether the individual responses reflect the overall perception of acceptability.
(2) Factors contributing to, or detracting from, acceptability, by assessing correlation between acceptability (AIM) and both appropriateness (IAM) and feasibility (FIM), as well as the TFA general acceptability measure and composite average score.

A further battery of questions was posed as part of the survey giving a greater degree of granularity to the assessment of acceptability, and in particularly sustainability, and analysed in the manner above (see Table 9, Supplemental Digital Content).

Statistical analysis was performed using R v4.0.3 (2020) (The R Foundation, IN, USA) and RStudio v2023.12.1+402 (2023) (MA., USA). Stacked bar charts were prepared using Microsoft Excel v16.81 (2024) (WA, USA).

## Results

### Patient recruitment & characteristics

**Fig. 1:**
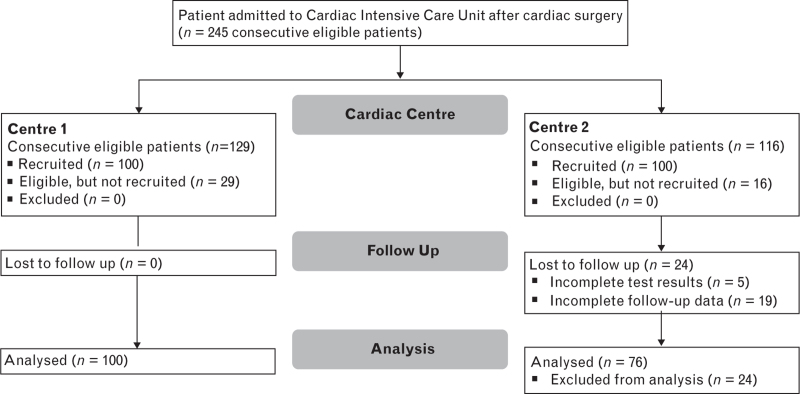
CONSORT diagram demonstrating flow through study.

The baseline survey was conducted at Centre 1 between January and February 2021, examining 25 consecutive adult patients undergoing cardiac surgery. At Centre 1, between July and September 2021, 100 patients from 129 eligible patients underwent urinary biomarker testing: a recruitment rate of 1.4 patients per day, where the maximum possible was 1.8 patients per day. The project ran at Centre 2 between January and February 2022 with a recruitment rate of 2.0 patients per day (Fig. 1).

### Biomarker testing and renal care bundle delivery

The urinary biomarker was elevated above the threshold for receiving the renal care bundle in 61 (34.7%) of patients overall. The median [IQR] biomarker concentrations were 0.15 [0.06 to 0.44] (ng ml^−1^)^2^ 1000^−1^ for the overall cohort, 0.08 [0.04 to 0.16] (ng ml^−1^)^2^ 1000^−1^ at Centre 1, and 0.37 [0.15 to 1.29] (ng ml^−1^)^2^ 1000^−1^ at Centre 2 (Table 4, Supplemental Digital Content).

**Fig. 2:**
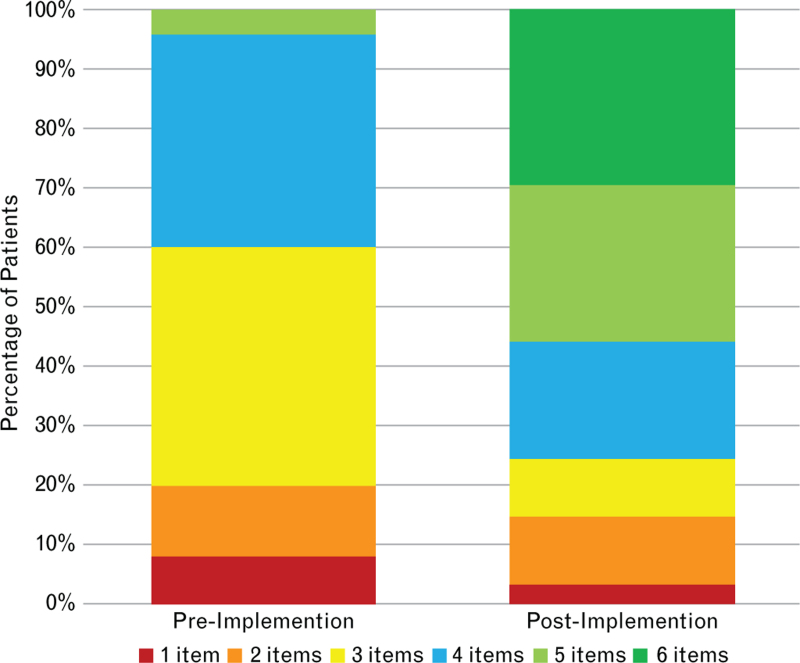
Stacked bar chart showing the percentage of patients receiving number of bundle items before and after implementation.

**Fig. 3:**
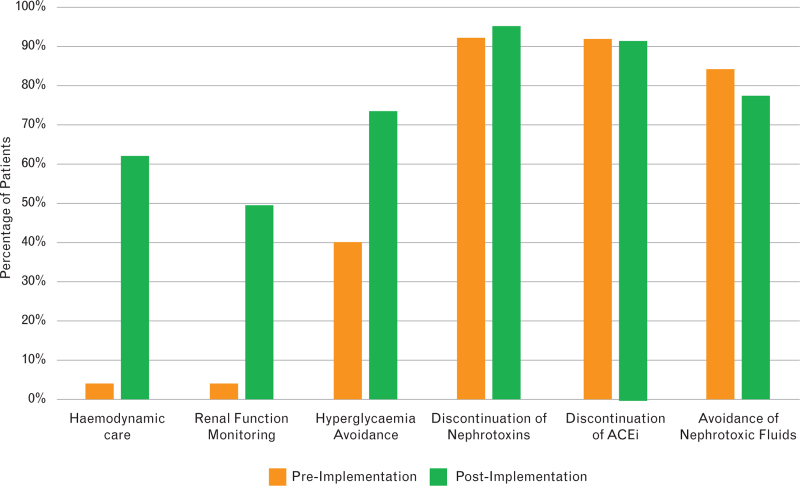
Clustered bar chart showing the percentage of patients receiving each element of the care bundle before and after implementation.

Before the innovation project, patients received a mean number of 3.36 ± 0.81 bundle items with none receiving all six items as standard care. Postimplementation, patients with an elevated biomarker value received a mean of 4.43 items (SD 1.45), with 29.5% (*n* = 18) receiving all six items. Care bundle delivery is shown in Table 5, Supplemental Digital Content and Figs. 2 and 3.

### Patient outcomes

Key clinical outcomes are detailed in Table 3.

**Table 3:** Key patient clinical outcomes across both sites

Clinical outcomes			Participants (*n* = 176)
AKI	In 72 h		53 (30.1)
	Stage	No AKI	123 (69.9)
		1	43 (24.4)
		2	5 (2.8)
		3	5 (2.8)
	Severe^a^		10 (5.7)
	Required RRT		7 (4.0)
LOS (days)	ICU		5 [3 to 6]
	Hospital		11 [8 to 15]

Data are *n* (%) or median [IQR].

a Severe AKI defined as Stage 2 or 3 KDIGO-defined AKI (by serum creatinine or urinary output criteria).

AKI, acute kidney injury; ICU, intensive care unit; LOS, length of stay; RRT, renal replacement therapy.

### Implementation survey respondents

Of the respondents, 49 completed the questionnaire (44% of all nursing/medical staff that were invited to respond), which included 8 medical/surgical consultants and 41 nursing staff. Eleven incomplete responses were excluded.

### Weiner Acceptability Metrics

**Fig. 4:**
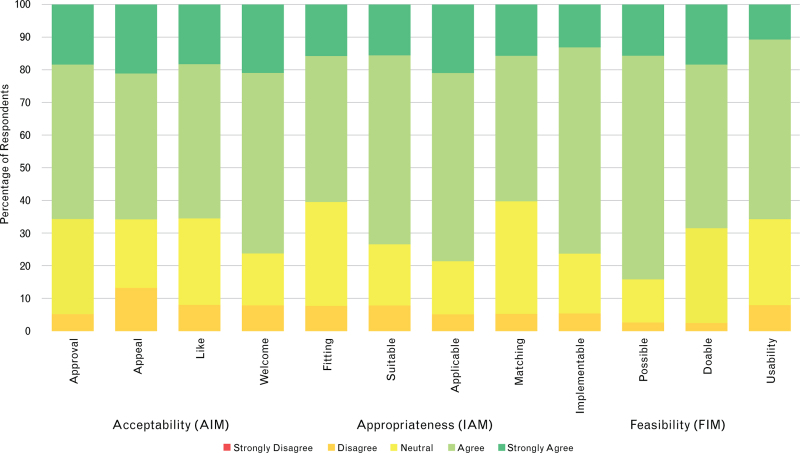
Stacked bar charts showing the proportion of each response to the Weiner Acceptability Metric Questionnaire.

The mean values for acceptability (AIM), appropriateness (IAM) and feasibility (FIM) were all between 3.6 to 4.0, indicating positive responses, with composite mean scores of 3.8 for all three domains. Median scores were four for all individual questions and composite scores, indicating positive responses. The full responses are shown in Table 6, Supplemental Digital Content and represented graphically in Fig. 4. AIM and IAM showed very strongly positive correlation (*R* = 0.85, *P* < 0.001), whilst AIM and FIM showed strongly positive correlation (*R* = 0.79, *P* < 0.001).

### Theoretical Framework of Acceptability Questionnaire Metrics

**Fig. 5:**
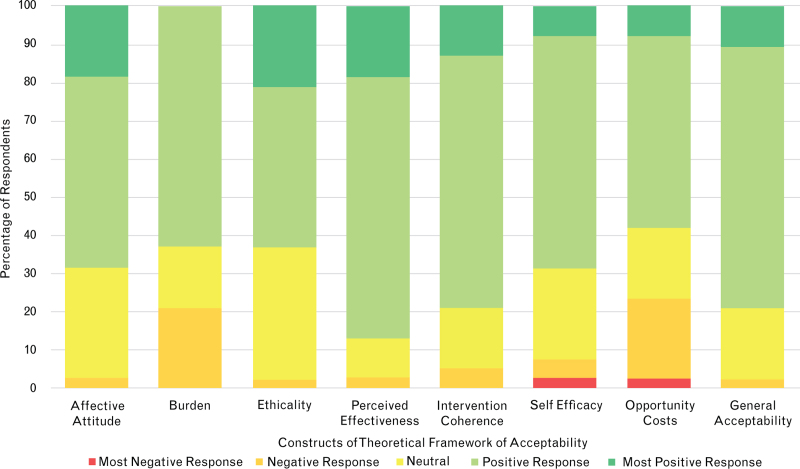
Stacked bar chart showing the proportion of responses to the constructs in the Theoretical Framework of Acceptability.

**Fig. 6:**
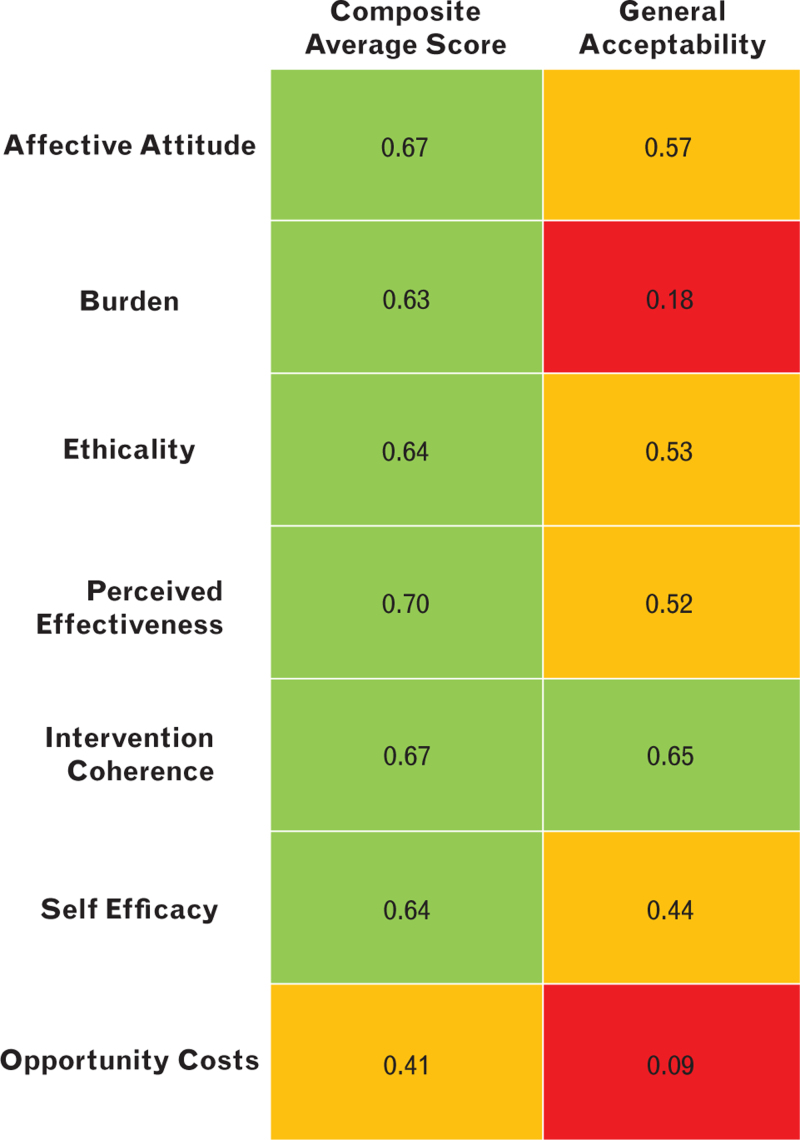
Correlation Matrix of Theoretical Framework of Acceptability Constructs with the Composite Average Score and General Acceptability (using Spearman's rank).

Most individual constructs scored a mean of between 3.6 and 4.0. The mean scores for the constructs of burden (3.4) and opportunity costs (3.4) were the lowest, although still in the positive response range. The perceived effectiveness score was highest (4.0), whilst general acceptability was 3.9 and the composite mean score was 3.7. All construct median scores were 4, indicating a positive response. All responses are shown in Table 7, Supplemental Digital Content and represented graphically in Fig. 5. Correlation was assessed between the individual constructs of the TFA and both the composite average score and the General Acceptability Score (Fig. 6).

The composite average score and general acceptability showed strong positive correlation (*R* = 0.65, *P* < 0.001) (Fig. 1, Supplemental Digital Content).

### Interaction of Weiner Acceptability with Theoretical Framework of Acceptance

General Acceptability in Sekhon's TFA showed a strong positive correlation with Weiner's Acceptability (AIM) (*R* = 0.75, *P* < 0.001). Appropriateness (IAM) demonstrated very strong correlation with Weiner Acceptability (AIM) (*R* = 0.85) (Table 8, Supplemental Digital Content and Fig. 2, Supplemental Digital Content).

## Discussion

In this study, we used two recent validated frameworks for the assessment of acceptability of the novel intervention of early urinary biomarker assessment after cardiac surgery and application of an appropriate AKI prevention bundle.

Medical, surgical and nursing staff reported positive responses for AIM, IAM and FIM. General acceptability according to Sekhon's TFA was also positive, with the perceived effectiveness construct contributing strongly to this outcome.

The findings of the PrevAKI and PrevAKI-Multicenter RCTs demonstrated that a renal biomarker-guided care bundle can reduce the incidence of severe (Stage 2–3) AKI in 72 h after cardiac surgery (28% vs. 5.7%, *P* < 0.001) and this evidence-based intervention bundle has been recommended in recent guidelines and consensus documents.^17,18,20,21^

In our study, implementation resulted in a higher delivered mean number of interventions intended to improve renal care (i.e. the renal care bundle items; 4.43 vs. 3.36), whilst the incidence of Stage 2/3 AKI in this study was lower than that observed in the PrevAKI and PrevAKI-Multicenter studies (5.7% vs. 29.7% and 14%, respectively).^20,21^

The primary outcome of this study was an assessment of the acceptability of an intervention that has been shown to have a clinically relevant outcome benefit, outside of strict research settings. This required the use of validated metrics for statistical assessment, but can also be assessed by looking at the rates of patient recruitment. Whilst reasons for patients not being assessed by urinary biomarker (e.g. bedside nursing/medical staff overlooked testing) were not formally collected, the rate of recruitment at both centres exceeded 70%, which should be considered successful for a new test, which required clinical staff to collect samples at a specific timepoint and provide interventions.

The implementation assessment can also be informed by the uptake of the bundle items. Our baseline survey suggested that patients received a mean of 3.36 items as part of routine care, which increased to 4.43 items after biomarker assessment. Similarly, the modal number of bundle items increased from 3 to 6 items. A multinational observational study analysing adherence to the KDIGO recommended elements of the renal care bundle after adult cardiac surgery similarly demonstrated that patients received a mean of 3.4 items in routine clinical practice. In that study, 5.3% of patients were receiving all bundle elements, whereas none were in our baseline survey.^24^

The Weiner Acceptability Metrics suggested a high degree of acceptability (AIM), appropriateness (IAM) and feasibility (FIM). The WAM results, reviewing the highest scoring items for each domain, suggest that staff ‘welcome’ innovation, find it ‘applicable’ (and therefore appropriate) and, as suggested by the rate of recruitment and testing, that it is ‘possible’. Whilst there were no overtly negative responses, the main detractors to each domain were ‘appeal’ (to acceptability), ‘fitting’ (to appropriateness) and ‘ease’ (for feasibility). The AIM and IAM demonstrated a very high degree of correlation, which suggests that there is a high degree of overlap between these two concepts, which is in keeping with much current thinking in implementation science.^22,25^ FIM showed strong correlation with AIM (*R* = 0.79, *P* < 0.001), suggesting that feasibility contributes to acceptability, but that these may not be overlapping constructs.

Considering the TFA, all of the individual constructs scored positively towards the intervention. ‘Perceived effectiveness’ scored highest (4.0), suggesting that the existing RCT evidence and logical coherence of the intervention increase the likelihood that staff will accept it. On the other hand, ‘burden’ and ‘opportunity costs’ scored lowest (both 3.4), suggesting that the introduction of a new intervention increases staff workload and may result in less time for other clinical tasks. However, the mean values did not suggest that respondents found this to be prohibitive. Our assessment of content validity (comparing the composite average score and the ‘general acceptability’) found that there was a high degree of internal validity.

The Weiner metrics IAM and FIM positively correlated with Sekhon General Acceptability (*R* = 0.71 and *R* = 0.62), but not overly positively, suggesting that these concepts contribute, but do not necessarily overlap. This reinforces that clinical staff will likely consider feasibility and appropriateness as part of the assessment of acceptability. Overall, this interpretation of the individual constructs and domains could be used to identify where to further strengthen future attempts to implement this technology-intervention bundle into clinical practice, emphasising the evidence base of the intervention, whilst devising strategies to limit time consumption.

Despite positive acceptability metrics, the ability to implement this technology-intervention bundle will require favourable business case assessments by individual institutions, comparing current costs to a potential shorter length of stay and lower requirement for ongoing renal care, albeit with the increased cost of the tests. The cost effectiveness of this intervention is likely promising, one of the key findings of the BigpAK study, but it remains to be comprehensively assessed.^26,27^

The key strength of this study is the use of novel implementation assessment of global acceptability, Sekhon's TFA, in addition to the more commonly used WAM. We have used this framework to analyse the implementation of an evidence-based intervention, providing insight into what facets support or impede universal adoption. Furthermore, it provides a template for this group, as well as the wider peri-operative community, as to the positive factors (perceived effectiveness) driving implementation, and those that will require addressing (opportunity costs and burden), alongside the particular bundle items which need further support (haemodynamic care and renal function monitoring).

The Sekhon TFA can be applied in other peri-operative settings to assess the acceptability of evidence-based best clinical practice and therefore potentially improve its implementation, for example, enhanced recovery bundles in obstetrics or general surgery.^1,2^ The TFA has been used in primary care, but to the best of our knowledge, this is the first time it has been used in peri-operative medicine.^28^

Furthermore, conducting our study in two centres, one an adult cardiac surgical centre and the other also an advanced heart failure centre, with significantly different median biomarker values and different requirements for bundle delivery (Table 4, Supplemental Digital Content) enabled an assessment across the spectrum of workload.

### Limitations

Our study is somewhat limited by the response rate of 44%, although this is higher than the median response rate of 37% in most anaesthetic studies.^29^ This may be explained by its conduct as an online questionnaire. A degree of bias entering the survey cannot be excluded, including sampling and selection or recall bias, which may increase with the smaller sample size, particularly with regards to the number of physicians. Participants were not screened for conflicts of interest.

The survey was also identical for all respondents, although each participant's interactions with the biomarker assessment or bundle delivery will be different, for example, for a consultant surgeon compared with a bedside nurse. However, all eligible participants were trained in biomarker assessment and bundle delivery. Furthermore, there was no specific interpretation of the results in the context of the number of patients each respondent had cared for, or the awareness of the biomarker result or clinical outcome of the patient. As an implementation science study, the treating clinicians were not blinded to any results (both biomarker and AKI status), and in this way it is reflective of ‘real-world’ conditions. Similarly, whilst participants were not explicitly told the cost of the biomarker test, they were aware that it was likely to be significantly higher than serum creatinine assays, as was discussed in the findings of a recent health technology assessment [NICE Diagnostics Guidance 39, June 2020].

We did not specifically collect information as to why some bundle items were not delivered, or indeed why some eligible patients did not undergo biomarker assessment, as comprehensively collecting the implementation metrics as presented here required a large survey, and a compromise to achieve reasonable response rates was made. This was particularly important as the survey may have failed to capture the opinions of those respondents who did not provide a number of the bundle items. Furthermore, while all patients should be receiving all elements of the renal care bundle, evidence suggests this is not the state of current practice, and a method of triaging efforts to the highest-risk patients remains valid.^24^ Implementation science studies may prove pivotal in understanding and addressing the reasons underlying incomplete bundle delivery.

The use of certain questions, particularly within the AIM domain, such as those relating to approval and appeal, can appear overtly personal. However, as part of a holistic assessment as to why implementation may be limited these were important factors to explore. The clinical use of the WAM was in its relative infancy when it was selected as an outcome measure for this study, given its specific development for healthcare. The TFA-Q was particularly novel, and provides a multifaceted and robust assessment of implementation, and we would advocate for its use in further peri-operative implementation science studies.

In conclusion, we have demonstrated that a biomarker-guided quality improvement treatment bundle, which was identified as best clinical practice in recent guidelines to improve kidney function after cardiac surgery, is deliverable outside of a research setting. The clinical team found it to be acceptable, appropriate and feasible, as assessed by comprehensive adoption and novel acceptability tests (WAM and TFA).

## Supplementary Material

Supplemental Digital Content
