# Supplementary material for: Adoption of a novel biomarker-guided quality improvement treatment bundle for patients with subclinical acute kidney injury after cardiac surgery: An implementation study
Source: Eur J Anaesthesiol. 2025 Nov 21;43(5):423–33. doi: 10.1097/EJA.0000000000002315 (PMC13048310; doi:10.1097/EJA.0000000000002315)

**Supplementary Table 1** KDIGO-Based renal care bundle, delivered to patients with elevated urinary biomarker ([TIMP-2]x[IGFBP7]) concentrations

| Discontinuation of nephrotoxic agents for 72h |
| --- |
| Use of advanced haemodynamic monitoring with algorithm-based optimization (See Supplementary Material) for 72h (or until deemed clinically stable enough to de-escalate invasive arterial monitoring) |
| Close renal functional marker monitoring: twice-daily serum creatinine measurement, hourly urine output and twice daily fluid balance monitoring for 72h |
| Avoidance of hyperglycaemia (defined as blood glucose >10mmol/L for >3h) for 72h |
| Discontinuation of ACEi and ARBs for 48h |
| Avoidance of HES, gelatin or chloride-rich solutions |

ACEi – angiotensin-converting enzyme inhibitors; ARB – angiotensin receptor blockers; HES – hydroxyethyl starch

**Supplementary Table 2** Patient characteristics

| **Characteristics** | | **Participants *(n=176)*** |
| --- | --- | --- |
| Age, Mean ± SD | | 64.1 ± 12.1 |
| BMI, Mean ± SD | | 28.0 ± 5.0 |
| Male, n (%) | | 132 (75) |
| DM, n (%) | NIDDM | 42 (23.9) |
|  | IDDM | 13 (7.4) |
| COPD, n (%) | | 9 (5.1) |
| sCr, Mean ± SD | | 86.3 ± 28.5 |
| Hb, Mean ± SD)* | | 131 ± 18.4 |
| Prev CS, n (%) | | 11 (6.3) |
| EF, n (%) | Normal* | 140 (80.9) |
|  | Mild | 16 (9.2) |
|  | Moderate | 15 (8.7) |
|  | Poor | 2 (1.2) |

*Missing data (complete values analysed)

BMI – Body Mass Index; COPD – Chronic Obstructive Pulmonary Disease; CS – Cardiac Surgery; DM – Diabetes Mellitus; EF – Ejection Fraction; Hb – Haemoglobin; IDDM – Insulin-Dependent Diabetes Mellitus; NIDDM – Non-Insulin Dependent Diabetes Mellitus; sCr – Serum Creatinine; SD – Standard Deviation

**Supplementary Table 3** Operative characteristics

| **Operative Characteristics** | | **Participants *(n=176)*** |
| --- | --- | --- |
| Procedures, n (%) | CABG | 95 (54.0) |
|  | AVR | 30 (17.0) |
|  | MVR | 23 (13.1) |
|  | CABG-Valve | 12 (6.8) |
|  | Aortic | 11 (6.3) |
|  | Complex | 16 (9.1) |
|  | Other | 10 (5.7) |
|  | Ablation | 5 (2.8) |
| Off-pump*, n (%) | | 25 (14.5) |
| Emergency/Urgent, n (%) | | 92 (52.3) |
| Time *min*, Median [IQR] | CPB* | 100 [84 to 122] |
|  | Cross-clamp* | 71 [53 to 91] |
| Temperature *°C*, Median [IQR] | | 33 [32 to 34] |
| CV Support, n (%) | Inotropes | 17 (9.7) |
|  | Vasopressors | 133 (75.6) |

*Missing data (complete values analysed)

AVR – Aortic Valve Replacement; CABG – Coronary Artery Bypass Graft; CPB – Cardiopulmonary Bypass; CV – Cardiovascular; IQR – Interquartile Range; MVR – Mitral Valve Replacement

**Supplementary Table 4** Urinary Biomarker ([TIMP-2]x[IGFBP7] (ng ml^-1^)^2^ 1000^-1^) Results

|  | **Centre 1 *(n=100)*** | **Centre 2 *(n=76)*** | **Overall *(n=176)*** | ***P*-value** |
| --- | --- | --- | --- | --- |
| Median [TIMP-2]x[IGFBP7] Concentration *(ng ml^-1^)^2^ 1000^-1^* [IQR] | 0.08 [0.04 to 0.16] | 0.37 [0.15 to 1.29] | 0.15 [0.06 to 0.44] | <0.001 |
| Elevated Biomarker Value ([TIMP-2]x[IGFBP7] ≥ 0.3 (ng ml^-1^)^2^ 1000^-1^, n (%) | 16 (16) | 45 (59.2) | 61 (34.7) | <0.001 |

**Supplementary Table 5** Postoperative Care Bundle Items & Delivery

|  | | **Overall *(n=61)*** |
| --- | --- | --- |
| Bundle of items administered, n (%) | 0 | 0 |
|  | 1 | 2 (3.3) |
|  | 2 | 7 (11.5) |
|  | 3 | 6 (9.4) |
|  | 4 | 12 (19.7) |
|  | 5 | 16 (26.2) |
|  | 6 | 18 (29.5) |
|  | Mean (SD) | 4.43  (1.45) |
| Haemodynamic care, n (%) | Monitoring | 41 (73.3) |
|  | Optimisation | 39 (63.9) |
|  | Total | 38 (62.3) |
| Renal Functional Monitoring, n (%) | Fluid Balance | 61 (100) |
|  | 12 hourly sCr | 30 (49.2) |
|  | Total | 30 (49.2) |
| Hyperglycaemia Avoidance, n (%) | | 41 (73.3) |
| Discontinuation of Nephrotoxins for 72h, n (%) | | 58 (95.1) |
| Discontinuation of ACEi/ARB for 48h, n (%) | | 56 (91.8) |
| Avoidance of Cl/Starch-based fluids, n (%) | | 47 (77.0%) |

ACEi – Angiotensin Converting Enzyme inhibitors; ARB – Angiotensin Receptor Blockers; Cl – Chloride-rich; sCr – Serum Creatinine; SD – Standard Deviation

**Supplementary Table 6** Wiener Acceptability Metrics (WAM) for survey respondents by site and combined.

| **Domain** | **Statement** | **Centre 1** | | **Centre 2** | | **Overall** | |
| --- | --- | --- | --- | --- | --- | --- | --- |
|  |  | **Mean ± SD** | **Median [IQR]** | **Mean ± SD** | **Median [IQR]** | **Mean ± SD** | **Median [IQR]** |
| **AIM** | The urinary biomarker test and renal care bundle meet my ***approval*** | 4.2 ± 0.6 | 4 [4 to 5] | 3.5 ± 0.8 | 3 [3 to 4] | 3.8 ± 0.8 | 4 [3 to 4] |
|  | The urinary biomarker test and renal care bundle are ***appealing*** to me | 4.1 ± 0.9 | 4 [4 to 5] | 3.4 ± 0.9 | 4 [3 to 4] | 3.7 ± 1.0 | 4 [3 to 4] |
|  | I ***like*** the urinary biomarker test and renal care bundle | 4.1 ± 0.7 | 4 [4 to 5] | 3.5 ± 0.9 | 4 [3 to 4] | 3.8 ± 0.9 | 4 [3 to 4] |
|  | I ***welcome*** the urinary biomarker test and renal care bundle | 4.2 ± 0.8 | 4 [4 to 5] | 3.6 ± 0.8 | 4 [3 to 4] | 3.9 ± 0.8 | 4 [4 to 4] |
| **AIM Composite Score** | | 4.2 ± 0.6 | 4.3 [2.8 to 4.5] | 3.5 ± 0.8 | 3.5 [3 to 4] | 3.8 ± 0.8 | 4 [3.3 to 4.3] |
| **IAM** | The urinary biomarker test and renal care bundle seem ***fitting*** | 4.0 ± 0.8 | 4 [4 to 4] | 3.4 ± 0.8 | 3 [3 to 4] | 3.7 ± 0.8 | 4 [3 to 4] |
|  | The urinary biomarker test and renal care bundle seem ***suitable*** | 4.1 ± 0.8 | 4 [4 to 5] | 3.6 ± 0.8 | 4 [3 to 4] | 3.8 ± 0.8 | 4 [3.3 to 4.0] |
|  | The urinary biomarker test and renal care bundle seem ***applicable*** | 4.3 ± 0.8 | 4 [4 to 5] | 3.7 ± 0.7 | 4 [3 to 4] | 4.0 ± 0.8 | 4 [4 to 4] |
|  | The urinary biomarker test and renal care bundle seem like a good ***match*** | 4.1 ± 0.8 | 4 [4 to 5] | 3.4 ± 0.8 | 3 [3 to 4] | 3.7 ± 0.8 | 4 [3 to 4] |
| **IAM Composite Score** | | 4.1 ± 0.7 | 4 [4 to 4.5] | 3.5 ± 0.7 | 3.5 [3 to 4] | 3.8 ± 0.8 | 4 [3.3 to 4.0] |
| **FIM** | The urinary biomarker test and renal care bundle seem ***implementable*** | 3.9 ± 0.8 | 4 [4 to 4] | 3.8 ± 0.6 | 4 [4 to 4] | 3.8 ± 0.7 | 4 [4 to 4] |
|  | The urinary biomarker test and renal care bundle seem ***possible*** | 4.2 ± 0.6 | 4 [4 to 5] | 3.8 ± 0.6 | 4 [4 to 4] | 4.0 ± 0.6 | 4 [4 to 4] |
|  | The urinary biomarker test and renal care bundle seem ***doable*** | 4.1 ± 0.9 | 4 [4 to 5] | 3.6 ± 0.6 | 4 [3 to 4] | 3.8 ± 0.8 | 4 [3 to 4] |
|  | The urinary biomarker test and renal care bundle seem ***easy*** to use | 4.0 ± 0.6 | 4 [4 to 4] | 3.4 ± 0.8 | 4 [3 to 4] | 3.7 ± 0.8 | 4 [3 to 4] |
| **FIM Composite Score** | | 4.1 ± 0.7 | 4 [4 to 4.5] | 3.7 ± 0.4 | 3.8 [3.3 to 4.0] | 3.8 ± 0.6 | 4 [3.5 to 4.0] |

**Supplementary Table 7** Responses to the constructs of the Theoretical Framework of Acceptability (TFA) by each centre and overall.

| **Construct** | **Centre 1** | | **Centre 2** | | **Overall** | |
| --- | --- | --- | --- | --- | --- | --- |
|  | **Mean ± SD** | **Median [IQR]** | **Mean ± SD** | **Median [IQR]** | **Mean ± SD** | **Median [IQR]** |
| Affective attitude | 4.1 ± 0.8 | 4 [3 to 5] | 3.7 (0.7 | 4 [3 to 4] | 3.8 ± 0.8 | 4 [3 to 4] |
| Burden | 3.4 ± 0.9 | 4 [3 to 4] | 3.4 ± 0.8 | 4 [3 to 4] | 3.4 ± 0.8 | 4 [3 to 4] |
| Ethicality | 4.2 ± 0.5 | 4 [4 to 4] | 3.5 ± 0.9 | 3 [3 to 4] | 3.8 ± 0.8 | 4 [3 to 4] |
| Perceived effectiveness | 4.2 ± 0.6 | 4 [4 to 5] | 3.9 ± 0.6 | 4 [4 to 4] | 4.0 ± 0.6 | 4 [4 to 4] |
| Intervention coherence | 4.1 ± 0.7 | 4 [4 to 4] | 3.7 ± 0.7 | 4 [4 to 4] | 3.9 ± 0.7 | 4 [4 to 4] |
| Self-efficacy | 3.6 ± 0.9 | 4 [3 to 4] | 3.7 ± 0.7 | 4 [4 to 4] | 3.7 ± 0.8 | 4 [3 to 4] |
| Opportunity costs | 3.5 ± 1.0 | 4 [3 to 4] | 3.3 ± 1.0 | 4 [3 to 4] | 3.4 ± 1.0 | 4 [3 to 4] |
| General acceptability | 4.2 ± 0.5 | 4 [4 to 4] | 3.6 ± 0.6 | 4 [3 to 4] | 3.9 ± 0.6 | 4 [4 to 4] |
| Composite Average Score | 3.9 ± 0.5 | 3.7 [3.4 to 4.3] | 3.6 ± 0.4 | 3.6 [3.3 to 3.9] | 3.7 ± 0.5 | 3.7 [3.3 to 4.0] |

IQR – Interquartile Range; SD – Standard Deviation

**Supplementary Table 8** Correlation Matrix of Weiner and TFA Metrics (Spearman’s, R)

|  | | TFA | | Weiner | | |
| --- | --- | --- | --- | --- | --- | --- |
|  |  | General Acceptability | Composite Average Score | AIM | IAM | FIM |
| TFA | General Acceptability |  |  | 0.75 | 0.71 | 0.62 |
|  | Composite Average Score |  |  | 0.77 | 0.60 | 0.61 |
| Weiner | AIM | 0.75 | 0.77 |  | 0.85 | 0.79 |
|  | IAM | 0.71 | 0.60 | 0.85 |  |  |
|  | FIM | 0.62 | 0.61 | 0.79 |  |  |

**Supplementary Table 9** Additional Responses to End of Phase Survey

| **Question** | **Scale** | **Mean ±** **SD Response** | **Median [IQR] Response** | **Missing or Non-Applicable Responses** |
| --- | --- | --- | --- | --- |
| When you use the urinary biomarker test and renal care bundle, how familiar do they feel? | 0 – Still feels very new  To  10 – Feels completely familiar  (Divided by 2 for analysis) | 2.3 ± 1.6 | 2.3 [1.0 to 3.5] | 2 |
| Do you feel the urinary biomarker test and renal care bundle are currently a normal part of your work? | 0 – Not at all  To  10 – Completely  (Divided by 2 for analysis) | 1.9 ± 1.7 | 1.8 [0.0 to 3.5] | 2 |
| Do you feel that the urinary biomarker test and renal care bundle will become a normal part of your work? |  | 3.1 ± 1.5 | 3.0 [2.4 to 4.1] | 2 |
| I can see how the urinary biomarker test and renal care bundle differ from usual ways of working | 5 - Strongly agree  4 – Agree  3 – Neither agree nor disagree  2 – Disagree  1 – Strongly disagree | 3.2 ± 0.8 | 3 [3 to 4] | 7 |
| Staff in this organisation have a shared understanding of the purpose of the urinary biomarker test and renal care bundle |  | 3.2 ± 0.9 | 3 [3 to 4] | 4 |
| I understand how the urinary biomarker test and renal care bundle affect the nature of my own work |  | 3.6 ± 0.8 | 4 [3 to 4] | 4 |
| I can see the potential value of the urinary biomarker test and renal care bundle for my work |  | 3.9 ± 0.9 | 4 [4 to 4] | 3 |
| There are key people who drive the urinary biomarker test and renal care bundle forward and get others involved |  | 3.7 ± 0.8 | 4 [3 to 4] | 3 |
| I believe that participating in the urinary biomarker test and renal care bundle delivery is a legitimate part of my role |  | 3.6 ± 0.9 | 4 [3 to 4] | 3 |
| I’m open to working with colleagues in new ways to use the urinary biomarker test and renal care bundle |  | 3.9 ± 0.9 | 4 [4 to 4] | 3 |
| I will continue to support the urinary biomarker test and renal care bundle |  | 4.0 ± 0.8 | 4 [4 to 4] | 3 |
| I can easily integrate the urinary biomarker test and renal care bundle into my existing work. |  | 3.7 ± 0.9 | 4 [3.5 to 4.0] | 3 |
| The urinary biomarker test and renal care bundle disrupt working relationships | 5 – Strongly disagree  4 – Disagree  3 – Neither agree nor disagree  2 - Agree  1 – Strongly agree | 3.6 ± 0.9 | 4 [3 to 4] | 3 |
| I have confidence in other people’s ability to use the urinary biomarker test and renal care bundle | 5 - Strongly agree  4 – Agree  3 – Neither agree nor disagree  2 – Disagree  1 – Strongly disagree | 3.5 ± 0.9 | 4 [3 to 4] | 3 |
| Work is assigned to those with skills appropriate to urinary biomarker test and renal care bundle |  | 3.4 ± 0.7 | 3 [3 to 4] | 4 |
| Sufficient training is provided to enable staff to implement the urinary biomarker test and renal care bundle |  | 3.2 ± 1.1 | 3 [2 to 4] | 4 |
| Sufficient resources are available to support the urinary biomarker test and renal care bundle |  | 3.3 ± 1.0 | 3 [3 to 4] | 3 |
| Management adequately supports the urinary biomarker test and renal care bundle |  | 3.4 ± 0.7 | 3 [3 to 4] | 5 |
| I am aware of reports about the effects of the urinary biomarker test and renal care bundle |  | 3.3 ± 1.0 | 4 [2.5 to 4.0] | 3 |
| The staff agree that the urinary biomarker test and renal care bundle are worthwhile |  | 3.5 ± 0.7 | 3 [3 to 4] | 3 |
| I value the effects that the urinary biomarker test and renal care bundle have had on my work |  | 3.7 ± 0.7 | 4 [3 to 4] | 3 |
| Feedback about the urinary biomarker test and renal care bundle can be used to improve them in the future |  | 4.1 ± 0.6 | 4 [4 to 4] | 4 |
| I can modify how I work with the urinary biomarker test and renal care bundle |  | 3.5 ± 0.9 | 4 [3 to 4] | 4 |

**Supplementary Figure 1** Scatter plots demonstrating degree of correlation (Spearman’s, R) between composite average score and general acceptability (at Centre 1, Centre 2 and Overall)


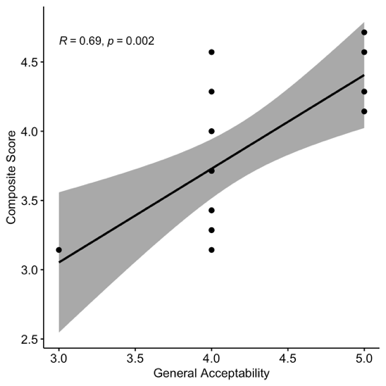

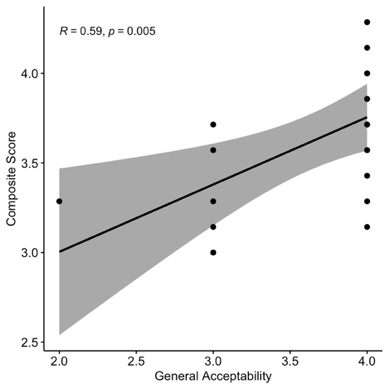

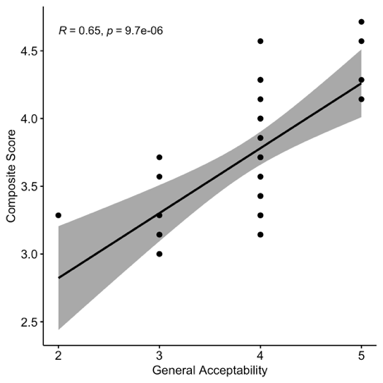


**Supplementary Figure 2** Scatter plots demonstrating correlation between Weiner Acceptability (AIM) and Theoretical Framework of Acceptability (TFA) General Acceptability and the Composite Average Score


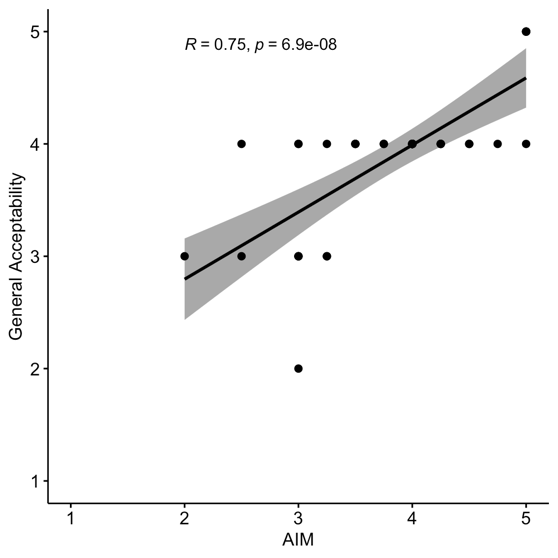

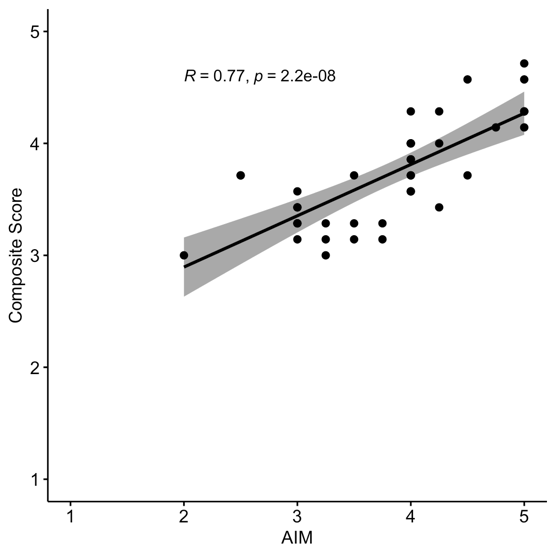

Supplement: Supplemental Digital Content [file ejanet-43-423-s001.docx]
